# Laccases as green and versatile biocatalysts: from lab to enzyme market—an overview

**DOI:** 10.1186/s40643-021-00484-1

**Published:** 2021-12-18

**Authors:** Tatiane Brugnari, Dayane Moreira Braga, Camila Souza Almeida dos Santos, Bruno Henrique Czelusniak Torres, Tatiani Andressa Modkovski, Charles Windson Isidoro Haminiuk, Giselle Maria Maciel

**Affiliations:** Biotechnology Laboratory, Department of Chemistry and Biology, Graduate Program in Environmental Science and Technology, Federal University of Technology, Paraná, Curitiba, Brazil

**Keywords:** Biocatalysts, Multi-copper oxidases, Ligninolytic enzymes, Immobilization, Patents

## Abstract

Laccases are multi-copper oxidase enzymes that catalyze the oxidation of different compounds (phenolics and non-phenolics). The scientific literature on laccases is quite extensive, including many basic and applied research about the structure, functions, mechanism of action and a variety of biotechnological applications of these versatile enzymes. Laccases can be used in various industries/sectors, from the environmental field to the cosmetics industry, including food processing and the textile industry (dyes biodegradation and synthesis). Known as eco-friendly or green enzymes, the application of laccases in biocatalytic processes represents a promising sustainable alternative to conventional methods. Due to the advantages granted by enzyme immobilization, publications on immobilized laccases increased substantially in recent years. Many patents related to the use of laccases are available, however, the real industrial or environmental use of laccases is still challenged by cost–benefit, especially concerning the feasibility of producing this enzyme on a large scale. Although this is a compelling point and the enzyme market is heated, articles on the production and application of laccases usually neglect the economic assessment of the processes. In this review, we present a description of laccases structure and mechanisms of action including the different sources (fungi, bacteria, and plants) for laccases production and tools for laccases evolution and prediction of potential substrates. In addition, we both compare approaches for scaling-up processes with an emphasis on cost reduction and productivity and critically review several immobilization methods for laccases. Following the critical view on production and immobilization, we provide a set of applications for free and immobilized laccases based on articles published within the last five years and patents which may guide future strategies for laccase use and commercialization.

## Introduction

Laccases (benzenediol oxygen oxidoreductase, EC 1.10.3.2) are enzymes capable of oxidizing a broad range of substrates, including ortho and para-diphenols, phenolic acids, aromatic amines and other electron-rich substrates, with concomitant reduction of molecular oxygen (O_2_) to water (Das et al. 2017; Brugnari et al. 2018). In addition, the oxidative action of laccases can be increased by the use of redox mediators in chemical reactions (Das et al. 2017; Yang et al. 2017a).

Laccases are mainly found in plants, fungi and bacteria and their variable biological functions may be correlated to the organism of origin, physiology and pathological conditions (Dwivedi et al. 2011; Deska and Kończak 2019). They are known for their intrinsic characteristics of high efficiency and sustainable applications with properties that make them suitable for green catalysis processes, including the synthesis of organic compounds and the treatment of environmental pollutants. Laccases are described in scientific literature as promising alternatives to conventional synthetic chemical processes considering their biodegradability and low or absent side reactions (Su et al. 2018; Fathali et al. 2019). Therefore, laccases are attractive tools for many different industrial processes with applications in pharmaceutical, textile, cosmetic, food, paper, and chemical (organic synthesis) industries.

Laccase has been increasingly studied and used as a green catalytic agent in production lines and, consequently, a high demand for commercial laccases is expected in the coming years. Hence, parameters optimization and scaling-up of processes for laccases production are essential aspects for further commercialization and application of the enzyme. Compared to the bench scale, the cost of laccases production can be reduced using industrial bioreactors, recombinant microorganisms, and alternative substrates such as agro-industrial wastes or by-products.

A variable productivity of laccases in different scales and conditions is often reported in the scientific literature (Liu et al. 2016; Deepa et al. 2020; Pinheiro et al. 2020; Abdelgalil et al. 2021). However, the analysis of laccase production costs or techno-economic analysis is mostly neglected, and few reported by authors. A decade ago, Osma and collaborators (2011) stated in their study that the real cost analysis of laccase production is an important feature for its industrial exploitation. Despite this conclusion, most authors simply define their cultivation methods or culture media as low cost, and in a few cases, an economic analysis of process feasibility has been presented (Pezzella et al. 2017; Hafid et al. 2021).

Besides, concerning laccases application, the use of soluble enzymes (including most laccases) is often limited by loss of enzyme activity which may be related to their low stability and/or performance under some operational conditions (Liu et al. 2018; Deska and Kończak 2019). These aspects impact the cost of most processes, especially on a large scale (Ba et al. 2012; Brugnari et al. 2018). To overcome these limitations, enzymatic immobilization techniques has been evaluated for maintenance or improvement of laccase catalytic properties. Immobilization also enables the reuse of the biocatalyst and increases operational stability, which consequently decreases the cost of using enzymes in biotechnological processes (Liu et al. 2018; Deska and Kończak 2019; Lassouane et al. 2019).

This review describes the structural and functional differences of laccases from bacteria, fungi and plants, the methods for laccase production, direct evolution, and molecular docking. Besides, scaling-up bioprocesses and the main techniques used for the immobilization of these enzymes are presented with a critical analysis of the advantages and disadvantages of each process. This paper ultimately reports an updated view on the use of free and immobilized laccases in several processes by emphasizing novel findings published in scientific literature and patents related to these green biocatalysts.

## Structure and mechanism of action of laccases

Several review articles have been published reporting discussions on laccases structure, functions, and mechanisms of action. Dwivedi et al. (2011), Arregui et al. (2019) and Janusz et al. (2020) detailed the structural and functional differences among laccases from fungi, bacteria and plants. The interpretation of the three-dimensional structure of laccases can be understood by Hakulinen and Rouvinen (2015), Chauhan et al. (2017) and Mehra et al. (2018). The enzyme structure and oxidation mechanism have been thoroughly described by Mot and Silaghi-Dumitrescu (2012) and Jones and Solomon (2015). These studies report the uniqueness and subtlety of these versatile biocatalysts. Laccases structural properties and mechanism of action are briefly described in the following sections.

**Fig. 1:**
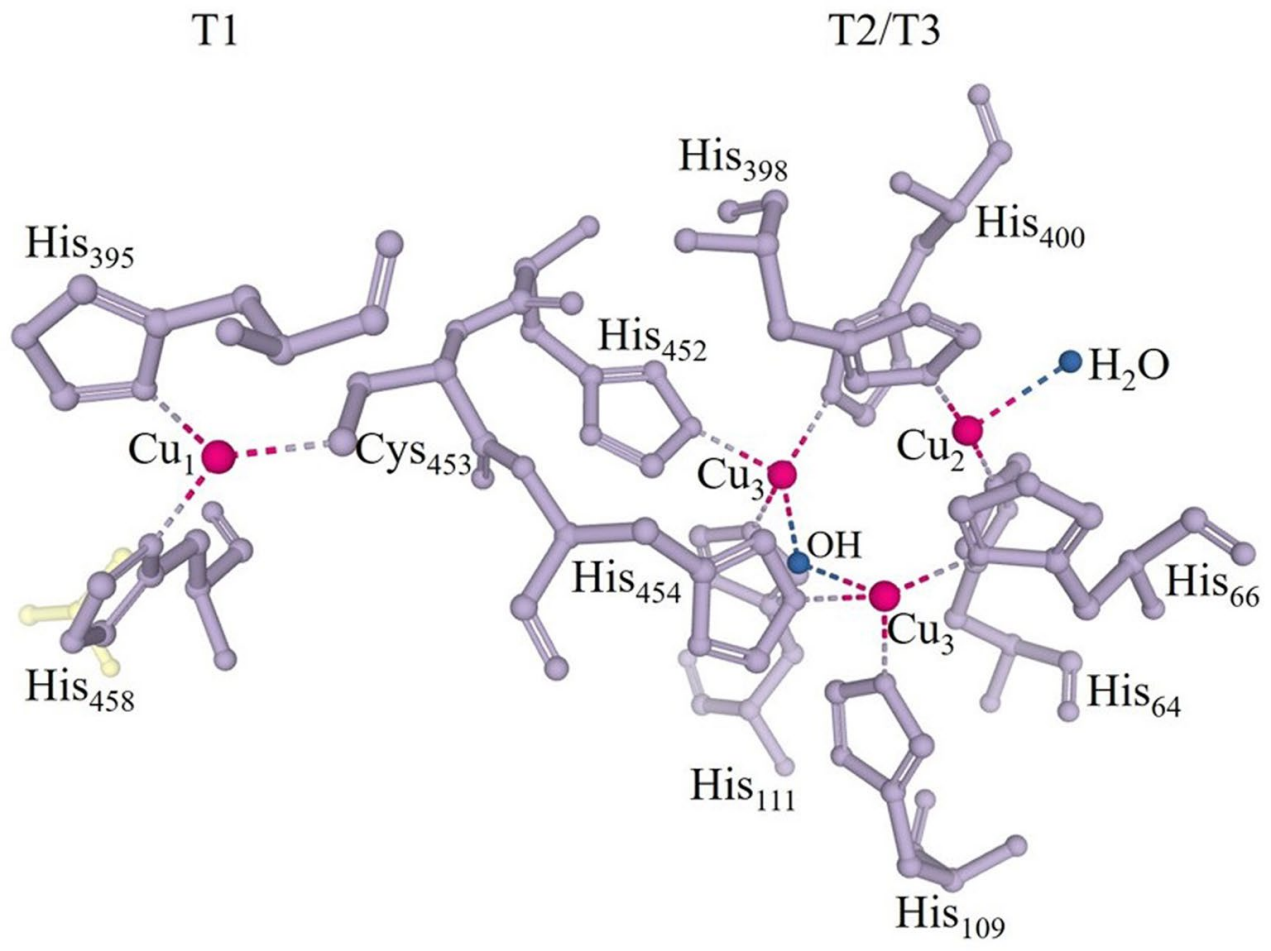
Representation of the copper catalytic site of *Trametes versicolor* laccases (RCBS—Protein Data Bank code 1GYC) coordinated with different amino acids; acronyms indicate: His—histidines, Cys—cysteíne. The copper atoms are represented by pink spheres

**Fig. 2:**
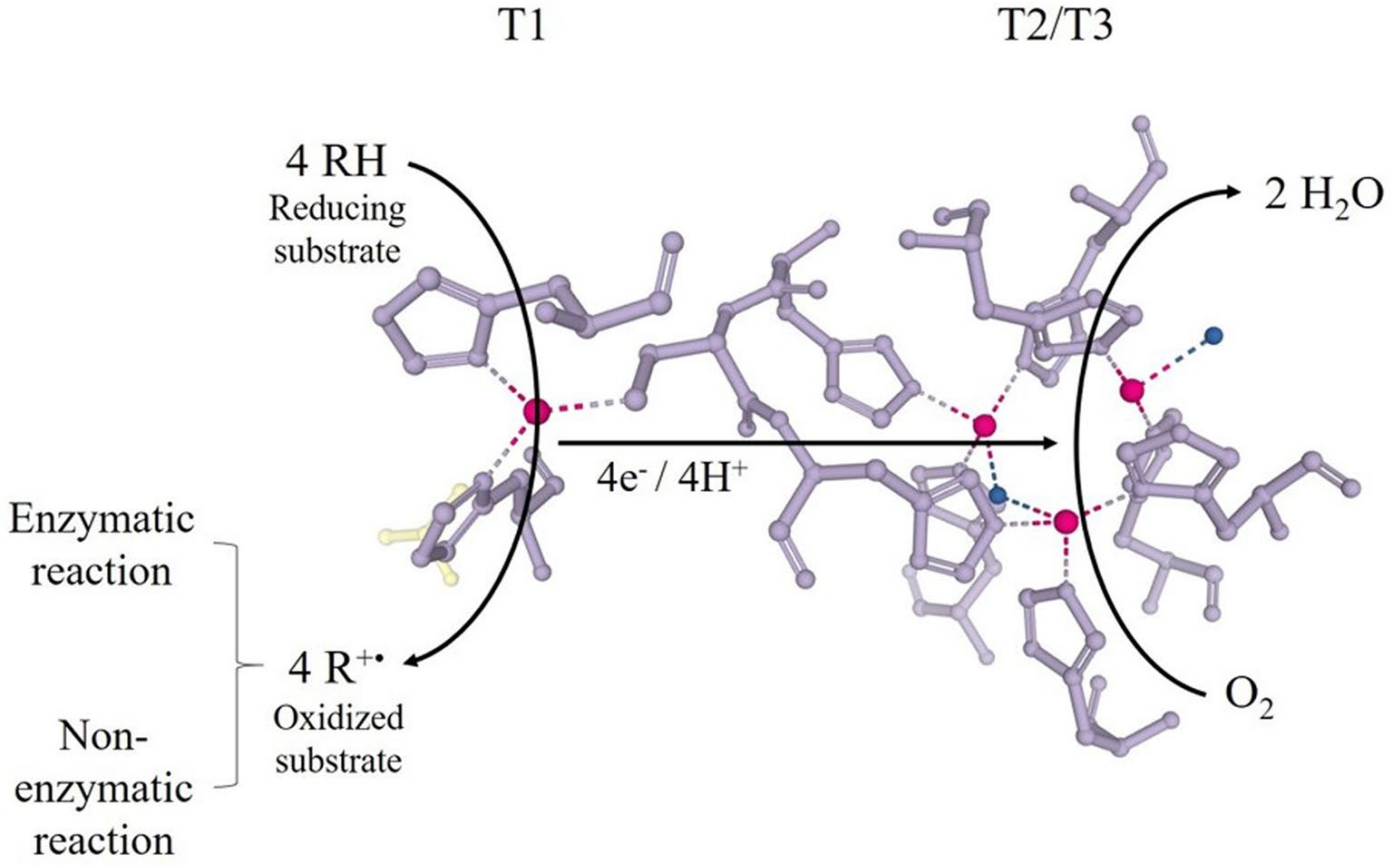
Representation of laccase mechanism of action in the catalytic center of *Trametes versicolor* (RCBS—Protein Data Bank code PDB 1GYC)

- Structure and properties of laccases
  Laccases (EC 1.10.3.2) are enzymes classified as multicopper oxidases, belonging to the superfamily of cupredoxin (Hakulinen and Rouvinen 2015; Arregui et al. 2019). Laccases are dimeric or tetrameric glycoproteins (Dwivedi et al. 2011; Reda et al. 2018; Deska and Kończak 2019) with a catalytic site typically containing four copper (Cu) atoms per molecule (Fig. 1). According to spectroscopic and paramagnetic properties, copper sites are categorized into three groups: Type-1 (one copper, T1), Type-2 (one copper, T2) and Type 3 (two coppers, T3) (Yang et al. 2017b; Arregui et al. 2019).
  Type-1 is the blue paramagnetic copper (Alcalde 2007) associated with the first stage of substrate oxidation (Arregui et al. 2019; Mot et al. 2020). This site has a trigonal orientation coordinated with a cysteine molecule and two histidines as conserved equatorial ligands (Fig. 1), and a variable axial ligand depending on the nature of the enzyme, usually methionine in bacteria and leucine or phenylalanine in fungal laccases (Hakulinen and Rouvinen 2015; Arregui et al. 2019). This structure has an absorption band at approximately 600 nm, characteristic of blue laccases (Dwivedi et al. 2011; Mot et al. 2020). Blue laccases represent typical laccases often reported in a variety of organisms, including *Pleurotus pulmonarius* (Marques De Souza and Peralta 2003), *Pleurotus ostreatus* (Palmieri et al. 1997), *Coriolus versicolor*, *Panus tigrinus*, *Phlebia radiata*, *Phlebia tremellosa* e *Agaricus bisporus* (Leontievsky et al. 1997), *Thermus* sp. (Navas et al. 2019) and *Trametes versicolor* (Agrawal and Verma 2020).
  Other laccases which do not present a typical absorption around 600 nm in T1 are known as white or yellow laccases (Mot et al. 2020). White laccases contain a copper ion, an iron ion and two zinc ions. In addition, they have an absorption band at 400 nm and different authors observed them in *Pleurotus ostreatus* (Palmieri et al. 1997), *Trametes hirsuta* (Haibo et al. 2009) and *Myrothecium verrucaria* (Zhao et al. 2014). The yellow laccases contain copper in a modified oxidation state and are described as a modification of the blue laccases (Dwivedi et al. 2011; Deska and Kończak 2019; Mot et al. 2020). They have been reported in *Panus tigrinus* (Leontievsky et al. 1997), *Daedalea flavida* (Sondhi et al. 2015), *Trametes* sp. (Wang et al. 2018), *Pleurotus ostreatus* (Pozdnyakova et al. 2006) *and Sclerotinia sclerotiorum* (Mot et al. 2020).
  Type-2 copper is paramagnetic and linked with two molecules of histidines and one of water, without absorption capacity in the visible spectrum (Dwivedi et al. 2011; Yang et al. 2017a). The Type-3 forms a diamagnetic binuclear center and shows absorption at 330 nm. Each copper in T3 is symmetrically coordinated with three histidine molecules and they are connected by a hydroxyl group that maintains the antiferromagnetic coupling between the pair of copper atoms (Alcalde 2007; Dwivedi et al. 2011). Together, the T2 and T3 copper sites form a trinuclear center (T2/T3) with 12 Å away from T1 (Alcalde 2007; Hakulinen and Rouvinen 2015). In addition, water results from the reduction of molecular oxygen ending the catalytic cycle of the enzyme (Alcalde 2007; Arregui et al. 2019).
  The structure of common laccases consists of three cupredoxin domains (Alcalde 2007; Hakulinen and Rouvinen 2015). The T1 site is in domain 3; the T2/T3 trinuclear center is inserted between domains 1 and 3, where are the residues that allow the coordination of copper atoms. Domain 2 is not involved in copper binding (Hakulinen and Rouvinen 2015) but it enables the formation of the trinuclear center and contains residues that contribute to the binding of the substrate at the enzymatic catalytic site (Alcalde 2007; Wong 2009). Laccases composed of two domains have also been reported in bacteria and are denominated as small laccases (Chauhan et al. 2017; Lisov et al. 2019; Olbrich et al. 2019). The copper-binding domains are conserved structurally, although the rest of the molecule exhibits variability. This conservation demonstrates a common mechanism of action of laccases (Alcalde 2007; Arregui et al. 2019).
  One of the most considerable properties of laccases is the redox potential (E^0^) of copper T1. This parameter represents the amount of energy needed to remove electrons from the reducing substrate at the beginning of the enzymatic catalysis (Arregui et al. 2019). According to E^0^ of copper T1, laccases are classified into high, medium and low redox potential. High and medium E^0^ laccases occur in fungi and laccases with low redox potential occur in bacteria and plants (Mate and Alcalde 2017; Deska and Kończak 2019). This potential difference is related to the binding residues in the axial position of copper T1. Hydrophobic residues (leucine or phenylalanine) are associated to high E^0^ of fungal laccases (Alcalde 2007).
- Mechanism of action
  Copper atoms are responsible for laccases catalytic action and mediate the oxidation–reduction process (Wong 2009). At rest, all copper ions are at the 2 + oxidation state (Yang et al. 2017a). In enzymatic catalysis, the first step is the oxidation of the substrate by mononuclear copper T1, which acts as an electron acceptor and changes Cu^2+^ to Cu^+^ oxidation state (Wong 2009). After removing an electron from the substrate, an unstable cationic radical is formed which can be oxidized by a second enzymatic reaction or undergo non-enzymatic reactions, such as hydration or polymerization (Alcalde 2007). The electrons extracted from the substrate at the T1 location are transferred to the T2/T3 center where the reduction of O_2_ to H_2_O occurs (Alcalde 2007; Wong 2009). For the complete reduction of molecular oxygen to water, four molecules of reducing substrate are needed (Alcalde 2007). Therefore, the stoichiometry of the enzymatic reaction is represented by the equation: 4RH + O_2_ → 4R + 2H_2_O, where RH represents the substrate (Wong 2009; Dwivedi et al. 2011). The mechanism of action of laccases is shown in Fig. 2.
  The catalytic efficiency of laccase in substrate oxidation depends on the difference in redox potential (ΔE^0^) between the substrate and the Cu T1 (Frasconi et al. 2010; Yang et al. 2017a). For substrates with E^0^ larger than those presented by the enzyme, low molecular weight compounds, known as redox mediators, are added to the reaction medium to expand the catalytic capacity of the laccase. Besides, when the substrate is too large to fit the enzyme active site, mediators are added to favor the oxidation of the substrate (Yang et al. 2017a). Commonly used mediators include natural ones such as syringaldehyde or synthetic ones such as 1-hydroxybenzotriazole (HBT) or 2,2′-Azino-bis (3-ethylbenzothiazoline-6- sulfonic acid (ABTS) (Yang et al. 2017a). Despite being efficient, the use of artificial mediators is limited by the high cost and toxicity associated with these substances. Thus, natural laccase mediators represent an economically and environmentally friendly alternative to expand biocatalytic applications (Cañas and Camarero 2010).
  Docking Simulation (MDS) is a simple and efficient tool that can be used to predict ligand–protein interactions. Through in silico experiments that integrate multiple data sources in molecular coupling simulations, it is possible to evaluate hundreds of thousands of orientations and conformations of a protein–ligand inside an enzyme active binding site and classify them according to their complex stability in terms of the estimated free binding energy (FEB) (Suresh et al. 2008; Cárdenas-Moreno et al. 2019).
  Although docking is an established tool for screening pharmaceutical drugs, its utility in predicting substrates that might be potential targets for laccase has only been explored in recent years. This is a faster alternative toward conventional methods when evaluating an already characterized enzyme. Since laccases are enzymes that can act upon a wide range of substrates and the substrate specificity differs from one laccase to another, the ability of laccases from different sources to biodegrade many different substrates can be investigated by MDS technique. Thus, in silico research is cost-effective for screening molecules susceptible to enzymatic degradation (Suresh et al. 2008). Researchers have used this approach to assess the potential of laccases to degrade recalcitrant pollutants, such as antibiotics (Cárdenas-Moreno et al. 2019), dyes (Agrawal et al. 2020; Pande et al. 2021), and others. In silico evaluation of laccase interaction with other proteins is also possible and was described by (Zaman et al. 2021), to understand the interactions of the estrogen receptor with laccase, which is known to have anticancer activity related to breast cancer cell lines as estrogen receptors play a vital role in the initiation and progression of breast cancer.

## Sources, production and directed evolution of laccases

- Fungal laccases
  Currently, in BRENDA database—The Comprehensive Enzyme Information System, more than 300 laccases are described, most of them from fungal species. In many fungi, laccase production is carried out by secondary metabolism when carbon and nitrogen sources in the growth medium are depleted (Yang et al. 2017a). Fungal laccases are related to sporulation processes, degradation of lignocellulose, formation of the fruiting body, pigment production and defense to stress in different fungal species (Alcalde 2007; Dwivedi et al. 2011; Góralczyk-Binkowska et al. 2020).
  A wide variety of fungi are reported to produce laccases and White-Rot Fungi (WRF) are the most recognized (Maciel et al. 2012; Arregui et al. 2019). *Pleurotus ostreatus* and *Trametes versicolor* are frequently reported in recent research on laccases (Alver and Metin 2017; de Araujo et al. 2017; de Freitas et al. 2017; Taheran et al. 2017; Brugnari et al. 2018; Patel et al. 2018; Rouhani et al. 2018; Fathali et al. 2019; Wen et al. 2019) and can be considered model organisms in basic and applied research for different purposes. However, other species are also potential laccase producers, including *Agaricus blazei* (Valle et al. 2015), *Flammulina velutipes* (Wang et al. 2015b), *Cerrena unicolor* (Zhang et al. 2018), *Trichoderma harzianum* (Ranimol et al. 2018), *Ganoderma lucidum* (Palazzolo et al. 2019), *Lentinus tigrinus* (Sadeghian-Abadi et al. 2019). In addition to the fungi from which laccases are commonly extracted, extremophile fungi are capable of producing laccases with great potential for industrial applications (Prakash et al. 2019).
  Fungal laccases are usually expressed in an extracellular form. However, some wood-degrading fungi can also produce intracellular laccases (Maciel et al. 2013; Arregui et al. 2019). These fungal enzymes present approximately 520–550 amino acids (Alcalde 2007) with some variations. For instance, *Flammulina velutipes* laccases expressed proteins containing 502–670 amino acids (Wang et al. 2015b). Recently, Pereira-Patrón et al. (2019), coded two *Trametes hirsuta* laccase genes with 521 amino acid sequences, demonstrating a high degree of identity (76–85%) with *T. villosa*, *T. versicolor* and other *T. hirsuta* strains. The authors also identified other characteristics, such as four copper sites and glycosylation sites, common to typical fungal laccases, such as those produced by *T. versicolor* (Piontek et al. 2002).
  The molecular mass of these enzymes can vary from 38 to 150 kDa (Manavalan et al. 2015). The isoelectric point is close to pH 4.0 (Baldrian 2006; Giardina et al. 2010), and consequently, they are more efficient in acid reactions with pH values between 3.5 and 5.5 (Martins et al. 2015). They express greater catalytic activity between 30 and 55 °C (Chandra and Chowdhary 2015), although some fungal laccases are more thermotolerant and can act between 25 and 60 °C (Shraddha et al. 2011).
  Fungal laccases comprise enzymes with medium and high redox potential (E^0^). Laccases of medium redox potential are commonly expressed in ascomycetes and basidiomycetes, with E^0^ ranging from 0.46 to 0.71 V (Shleev et al. 2004; Mate and Alcalde 2017). Other authors, however, classify this lower limit as laccases with low redox potential. Frasconi et al. (2010), for example, reported laccases of the ascomycete *Melanocarpus albomyces* with E^0^ = 0.46 V, classifying them as low redox potential. Therefore, this classification is not clearly defined.
  High potential redox laccases are synthesized by white-rot basidiomycete fungi, with E^0^ in copper T1 ranging between 0.73 and 0.79 V (Mate and Alcalde 2017). This characteristic gives them a greater interest in biotechnological applications, since they are able to oxidize substrates with high E^0^ (Martins et al. 2015). For example, in applications associated with bioremediation, fungal laccases have demonstrated potential to remove Bisphenol A (BPA) (de Freitas et al. 2017; Brugnari et al. 2018; Lassouane et al. 2019), whose E^0^ is above 600 mV (Arregui et al. 2019).
  Though laccases can be produced on laboratory scale with many different fungi in glass flasks or trays (usually in static cultures), few authors have attempted to study the production of fungal laccases in bioreactors such as stirred tanks or airlift (Table 1). Innovations in this field are quite uncommon in the scientific literature. Considering the industrial importance of laccases so that they are efficiently applied, research describing the production of these enzymes in large quantities at low cost is of great relevance, for example by optimizing the fermentation medium and using a technology that can be easily transferred to a pilot or industrial scale.
  Pinheiro et al. (2020) compared the production of laccases from *Trametes versicolor* in Erlenmeyer flasks, a BioFlo bioreactor and tray bioreactor, with agroindustrial wastes as substrate. The largest enzyme production was achieved with the BioFlo bioreactor (specific activity of 55. 24 U/mg of protein). Thus, expanding the enzyme production from a laboratory scale (Erlenmeyer flasks) to a larger scale production is feasible and essential for industrial use.
  Some important parameters to be evaluated for the cultivation of fungi in a bioreactor include morphological growth (formation of pellets in submerged/agitated cultures and mycelia in solid-state cultures), agitation, mass transfer, oxygenation, broth rheology, pH, temperature, mode of operation (batch or continuous) and the cost of culture media.
  Airlift or Stirred Tank (STR) bioreactors with low mechanical agitation, provide a low shear environment to produce fungal laccases, preventing friction and damage in mycelial morphology and allowing the pelletized growth of fungi and a satisfactory enzymatic production (Wang et al. 2016; Bettin et al. 2020).
  Production of laccases in bioreactors (except Erlenmeyer flasks) using solid-state fermentation (SSF) is much less reported in the scientific literature than submerged cultivation (Wang et al. 2019). In SSF, many different agroindustrial residues or byproducts can be used as substrates for laccase production, which is eco-friendly and reduces the cost of the process. However, SSF parameters such as pH, temperature, moisture, aeration and agitation (when possible) are difficult to control in a bioreactor and require the design of specific equipment for such proposal. Hence, scaling up the process of laccase production by SSF is still a laborious challenge.
  Liu et al. (2016) evaluated the production of laccase by a wild strain of *Pycnoporus* sp. SYBC-L3 in a pilot-scale 500-L STR and 5-ton STR bioreactor, with resulting enzymatic activities of 60 U/mL and 80 U/mL, respectively. The process cost evaluation for the 5-ton reactor showed that labor had a major contribution (50%) of the total cost in industrial production, and feedstock accounted for less than 5% of the total cost.
  Recently, Hafid et al. (2021) simulated the economic feasibility of producing laccase from *Pycnoporus sanguineus* with oil palm empty fruit bunch as substrate on an industrial scale (batch bio-refinery concept) using previously acquired laboratory-scale production data and specific software (SuperPro Designer). The authors predicted a laccase production cost of 14.26 US$/kg, and the highest contribution of capital (65.87%), followed by raw materials (31.68%), labor (2.17%), and other inputs (such as utilities and consumables).
  The difficulties often presented when scaling-up the production of laccases secreted from native sources, mainly because of low production yield and high cost of preparation/purification procedures, have promoted the number of research with recombinant laccases. Besides, native sources usually produce a mixture of isozymes, which may not be desirable for commercial purposes (Piscitelli et al. 2010; Preethi et al. 2020). Thus, heterologous expression emerged as a strong ally in this process, allowing the production of laccases with desired properties (such as different substrate specificities or improved stability) for industrial applications and providing high enzymatic yield/productivity (Piscitelli et al. 2010).
  The high redox POXA1b laccase from *Pleurotus ostreatus,* with a stable profile and relevant activity, was selected by Pezzella and collaborators (2017) as a marketable enzyme case study. POXA1b was produced heterologously using the yeast *Pichia pastoris*. The study was conducted in shake flasks and a bioreactor. The economic feasibility of producing recombinant laccase was simulated, describing the case of a small and medium-sized Italian company. Two variables were evaluated: (I) production based on an inducible methanol system; (II) production based on the constitutive system fed with glycerol. The study demonstrated that glycerol-based fermentation is more economical than methanol. The price calculated to produce rPOXA1b was €0.34 kU^−1^ for a glycerol-based process, very competitive with the price of commercial laccases when the study was carried out.
  Increased enzymatic productivity is achieved through the use of multiple copies of genes, strong promoters, and efficient signal sequences, properly designed to direct proteins to the extracellular medium, thus simplifying downstream processing (Piscitelli et al. 2010). Though the heterologous expression of laccases has proved to be advantageous and can be produced according to the need of the process, the industrial use of recombinant laccases is still unusual. A more detailed discussion of currently available commercial laccases including recombinant laccases is found in Sect. "Commercial laccases and patents".
  Preethi et al. (2020), published a comprehensive review focused on recombinant laccases and their engineering perspective, attesting to commercially available laccase cloning reports from a broad category of microbial sources (including fungi) and their biotech applications. Significant advances have been reported in this area, so far about 50 cloning and expression studies have been carried out on various laccase sources such as plants, bacteria, fungi, actinobacteria, and mushrooms using expression systems such as *E.coli* BL21, *E. coli* DH5 α, *E. coli* XL1, *Kluyveromyces lactis*, *Saccharomyces cerevisiae*, *Yarrowia lipolytica*, *Pichia pastoris*, *Aspergillus oryzae*, *Aspergillus soybeane* strain 1860, *Pichia methanolica* and transgenic corn.
  The use of cultivated substrate and biomass after enzyme extraction is also an interesting aspect and few explored by researchers. The production of laccase by microorganisms can generate a large amount of biomass, which is often discarded at the end of the fermentation process. Lú-Chau et al. (2018), after scaling-up the cultivation of *Ganoderma lucidum* for laccase production, analyzed the possibility of using biomass generated in a bioreactor (30L) for the extraction of Ganoderic acid, a powerful triterpenoid with several pharmacological activities, such as inhibitor of cholesterol synthesis, antihistamine, antihypertensive, antitumor, and antiviral. After cultivation of *G. lucidum*, 2.26 g L^−1^ of total biomass (mycelium and spores) was produced and the concentration of ganoderic acid extracted from the mycelium was 24.3 ± 4.4 mg g^−1^ of fungal biomass. The conventional production method of GAs is carried out by extracting this compound from the basidiome of the fungus; however, this process usually takes several months, and the yield is low.
- Bacterial laccases
  The first report of bacterial laccases occurred in *Azospirillum lipoferum*, isolated from rice rhizosphere (Givaudan et al. 1993). So far, most of the laccases characterized belong to *Bacillus* and *Streptomyces* genus. In *Bacillus*, laccases are part of the endospore coat, giving them brown pigmentation that protects bacteria from external stress and UV light (Martins et al. 2015). Laccases of the genus *Streptomyces* act on morphogenesis, sporulation, pigmentation, interaction between bacteria, production of antibiotics and, in addition, are able to degrade lignin (Janusz et al. 2020).
  Recently, Narnoliya et al. (2019) coded *Bacillus atrophaeus* laccases genes, corresponding to a protein with 278 amino acids. Phylogenetic analysis indicated a relationship between laccase genes from different *Bacillus* species. The protein structure analysis of *B. atrophaeus* laccases predicted amino acid residues (43Lys, 44Pro, 45Pro e 47Glu), which are involved in the substrate binding.
  In the last decade, several bacterial species have been reported in the production of laccases, including *Bacillus safensis* (Singh et al. 2014), *Bacillus tequilensis* (Sondhi et al. 2015), *Pseudomonas* sp. (Chauhan et al. 2017), *Geobacillus* sp. (Jeon and Park 2020), *Marinomonas mediterrânea* (Road 2020) and *Pseudomonas putida* (Karuna and Poonam 2020). Most laccases produced by bacteria are intracellular as in *B. subtilis*, *M. mediterranea*, *Sinorhizobium meliloti* and *Thermus thermophilus* (Janusz et al. 2020). On the other hand, extracellular laccases were found in some species of *Bacillus* (Dubé et al. 2008)*, Streptomyces* (Janusz et al. 2020) and *Brevibacterium halotolerans* N11 (KY883983) (Reda et al. 2018).
  Most of these enzymes are monomeric with molecular mass ranging from 50 to 70 kDa (Arregui et al. 2019). For bacterial enzyme production, the optimum temperature is close to 45 ºC (Muthukumarasamy and Murugan 2014) and the pH considered optimal can vary according to the substrate (Arregui et al. 2019). In enzymatic reactions, bacterial laccases have maximum activities at neutral or alkaline pH (Muthukumarasamy and Murugan 2014; Martins et al. 2015; Janusz et al. 2020).
  Bacterial laccases comprise enzymes with low redox potential (E^0^) from 0.4 to 0.5 V (Chandra and Chowdhary 2015; Mate and Alcalde 2017). Despite the low redox potential, these enzymes are more robust than fungal laccases in extreme conditions (Guan et al. 2018; Narnoliya et al. 2019) especially concerning their high thermal stability, significant activity in wide pH variation and for supporting high concentrations of sodium (Held et al. 2005; Guan et al. 2018), which are advantages for industrial applications. In addition, bacteria usually grow faster than fungi in liquid media which favor scaling-up processes for laccase production (Chandra and Chowdhary 2015; Chauhan and Jha 2018; Deepa et al. 2020).
  Although the period of bacterial growth is shorter compared to fungi, in general, these microorganisms present lower production of extracellular laccase (which implies in cell rupture as additional process for laccase production), thus requiring studies that focus on commercial level production and high-yield cultures in a shorter period.
  Deepa et al. (2020), recently isolated different strains of laccase-producing bacteria in the waste of soap industry. Among them, an isolate of *Bacillus* sp. BAB4151 produced large amounts of the enzyme. The laccase production process was optimized and scaled to a 5 L bioreactor. Initially, the optimization by OVAT (one variable at a time) showed a 2.9 times improvement in laccase yield, which increased even more by 1.4 times during scale-up in a bioreactor. The authors emphasize that the capacity of *Bacillus* sp. in producing laccase and retaining productivity during process scaling-up at the bioreactor level suggests that the organism may be of great commercial importance.
  The production of newly discovered bacterial extracellular laccase *Lysinibacillus macroides* LSO isolated from pulp and paper industry effluents was investigated in a 10 L stirred tank bioreactor under batch operation conditions (Abdelgalil et al. 2021). The highest productivity was achieved in the initial period of cultivation and laccase yield of 7653.2 UL^−1^ min^−1^ was observed in this study. The authors also explored the use of sugarcane bagasse as a cheap and accessible raw material for industrial scale production.
- Plant laccases
  Plant laccases were first reported in purified sap of the lacquer tree (known as *urushi*) from the *Rhus vernicifera* tree (Alcalde 2007). In plants, this enzyme plays important regulatory roles, such as the polymerization of lignin, the response to environmental stress, defense mechanism, wound healing, maintenance of the structure and the polymerization of phenolic compounds (Sharma and Kuhad 2008; Berthet et al. 2012; Wang et al. 2015a; Janusz et al. 2020). In addition, Bryan et al. (2016) suggested that laccases contribute to the integrity of the cell wall.
  Recently, laccase genes have been identified in different plant species, including *Gossypium* spp. (Balasubramanian et al. 2016), *Oryza sativa* (Liu et al. 2017), *Prunus avium* (Berni et al. 2019), *Pyrus bretschneideri* (Cheng et al. 2019), *Amborella trichopoda*, *Glycine max*, *Physcomitrella patens*, *Ricinus communis*, *Triticum aestivum*, *Vitis vinifera* (Liu et al. 2020), *Setaria viridis* (Simões et al. 2020) and *Zea mays* (Xie et al. 2020).
  Plant laccases are monomeric and extracellular (Solomon et al. 1996). Proteins are constituted of approximately 500–600 amino acids with higher molecular weight than fungal and bacterial laccases, between 60 and 130 kDa (Singh Arora and Kumar Sharma 2010; Wang et al. 2015a). They have an isoelectric point between 5.0 and 9.0 and optimum pH values between 5 and 7 (Berthet et al. 2012; Janusz et al. 2020), and thus maximum activities are achieved in neutral or alkaline pH (Arregui et al. 2019). These enzymes, like bacterial ones, have low redox potential, with E^0^ values below 0.46 V (Mate and Alcalde 2017).
  Both plant and fungal laccases are glycosylated enzymes, and the carbohydrate fraction is mainly composed of mannose, *N*-acetyl glucosamine and galactose. In plant laccases, the percentage of carbohydrates is higher, between 22 and 45%, while in fungal laccases, the extension of glycosylation represents 10–25% (Dwivedi et al. 2011).
  To date, studies involving laccases produced by plants have focused on the characterization and sequencing of genes, cloning and mutation for genetic improvement. There are no reports on the advantages and disadvantages of using plant laccases compared to fungal and bacterial laccases in reaction conditions or industrial applications. However, due to the role of plant laccases in lignin biosynthesis, Wang et al. (2015a) suggested a great potential in the use of plant laccases in power plants to improve the production of biofuels.
  Plant laccases have not been much explored for their use in systems that involve larger-scale production. Although not evaluated individually Watharkar et al. (2018) reported that laccases and other enzymes from *Asparagus densiflorus* can be efficiently applied in the treatment of industrial textile effluents. The phytodegradation of Rubin GFL dye (RGFL) and a real effluent was evaluated on a laboratory scale, using a vertical subsurface flow phytoreactor. The treatment of real effluent was also studied on a large scale, in a high-rate transpiration system (HRTS) through the planting of *A. densiflorus* beds. The expression of the laccase enzyme was high (178%) and *A. densiflorus* exhibited a good ability to degrade dyes, heavy metals and reduce the toxicity of effluents. In the field remediation study, 67% of the soil pollution was removed in 30 days.
- Directed evolution as a strategy for obtaining stable laccases
  The difficulty found for heterologous expression of fungal laccases is often a bottleneck for the application of these enzymes. Wild-type enzymes are often not robust enough for direct use in industrial applications and must be improved for better catalytic efficiency and stability. Targeted evolution is an innovative and versatile technology, applied from protein engineering, that assists in adapting these enzymes to perform specific desired functions. The directed evolution cycle involves iterative rounds of DNA library design and generation, gene expression and screening of enzyme library members. Multiple properties can be optimized in parallel and improved variants can be isolated, characterized and used as templates for further rounds of evolution (Bell et al. 2021).
  According to Mills et al. (1967) modification methods are mainly grouped into two categories, namely rational design methods and non-rational design methods. In non-rational design, the understanding of relationship between protein structure and function is not necessary. For example, error-prone PCR is a non-rational design method, which can perform directed evolution and selection of genes encoding stable enzyme molecules with higher specificity for a substrate. This method was used by Dai et al. (2021) in the development of a new bacterial laccase with high catalytic efficiency for dye degradation. This in vitro directed molecular evolution model can introduce large phenotypic differences through small sequence alterations, and target strains can be selected, which facilitates comparative sequence analysis. However, the researchers emphasize that error-prone PCR can only transform a small sequence into the original protein, and it is generally adequate for smaller gene fragments (< 2000 bp).
  *Saccharomyces cerevisiae* is the usual host for fungal laccase engineering. Directed evolution has helped to increase the secretion of active laccases by yeast, via modification in the native signal peptide is usually replaced by the preproleader of *S. cerevisiae* alfa mating factor (MFα1). During the directed evolution of *Pycnoporus cinnabarinus* laccase (PcL) for its functional expression in *S. cerevisiae*, several mutations accumulated in the evolved leader (α3PO) raised 40-fold the secretion of native PcL compared with the levels obtained with the native α leader (αnat) from Invitrogen (Camarero et al. 2012; Aza et al. 2021). Then, the evolved α3PO leader was further mutated successively, giving rise to the α9H2 leader, which differs in seven mutations (Aα9D, Aα20T, Qα32H, Fα48S, Sα58G, Gα62R, Aα87T) from the αnat leader. The α9H2 leader contributed to obtaining the highest yields reported so far for a basidiomycete laccase produced in *S. cerevisiae* (De Salas et al. 2019; Aza et al. 2021).
  Recently Aza et al. (2021) evaluated the secretion potential of the α9H2 leader as a signal peptide for the production in *S. cerevisiae* of laboratory-engineered laccases. Two laccases from *Agaricales fungi*, *Agrocybe pediades*, ApL (ID 823,363 JGI), and *Pleurotus eryngii*, PeL (ID 152,153 JGI) were assayed and, as in previous works with other basidiomycetes, their secretions increased after targeted evolution. In this research, the authors also evaluated and discussed the role of protein N-glycosylation in laccase production and properties, and found evidence that the addition of new N-glycosylation sites can stimulate the production of active and properly folded laccases by *S. cerevisiae*, especially when they are impaired in secretion due to aggregation. They also observed that the hyperglycosylation does not contribute to improving the stability of the enzyme.
  Although laccases with high fungal redox potential (HRPLs) are considered the most relevant for industrial and environmental applications among biocatalysts, their implementation has been delayed by the lack of thermostable variants. Using this premise and introducing a set of 27 stabilizing mutations identified in previous evolutionary campaigns focused on the HRPL of the basidiomycete PM1, Mateljak and Alcalde (2021) developed what appears to be the most thermostable HRPL reported to date. They developed a variant with a half-life of thermal inactivation at 75 °C of 225 min, 32-fold superior to that of the parental enzyme, and remarkable stability at both acidic and basic pHs.
  Interestingly, paleoenzymologists are bringing back protein sequences from long-extinct organisms to examine the events of natural molecular evolution. As reported by Gomez-Fernandez et al. (2020), the ancestral sequence reconstruction and resurrection provide significant information for protein engineering, yet its combination with directed evolution has been few explored. According to these researchers, studies in this field are highly relevant, since ancestral enzymes may be suitable in directed evolution experiments aimed at promoting the evolution of enzymes towards functions apparently never explored in nature or to revive former activities, as these enzymes were largely generic biocatalysts, which in turn had to adapt to the hostile environments of the ancient land (particularly those in the pre-Cambrian period) performing under greater stability.
  Several ancestral nodes of fungal laccases dating back from 500 to 250 million years were resurrected (Gomez-Fernandez et al. 2020). Unlike modern laccases, the resurrected Mesozoic laccases were readily secreted by yeast, with similar kinetic parameters, broader stability, and distinct pH activity profiles. The reactivated Agaricomycetes laccase carried 136 ancestral mutations, a molecular testimony to its origin, and it was challenged to directed evolution to improve the rate of 1,3-cyclopentanedione oxidation, a diketone initiator commonly used in vinylization/polymerization reactions.
  These studies shed light on the production of enzymes and opened new engineering opportunities that can also be transferred to other enzymatic systems.

**Table 1:** Fungal laccase production in bioreactors: cultivation conditions and enzymatic activity

Fungi	Bioreactor / agitation	Substrate	Dissolved oxygen / aeration	Cultivation time (days)	Laccase activity and extracellular protein concentration (mg / L)	References
*T.versicolor*	Air-lift (without mechanical agitation)	Potato dextrose broth / mineral solution / vanillic acid as inducer	0,85 vvm by continuous injection	7	14,902 U/L 142.46 mg/L	Wang et al. (2016)
*Pleurotus sajor-caju*	Stirred-tank (200 rpm)	Glucose / pure casein / CuSO_4_ / benzoic acid / mineral solution	30% of saturation	2.75	80,000 U/L 81 mg/L	Bettin et al. (2020)
*T. versicolor*	Stirred-tank under static condition	Cotton gin waste and vinasse / peptone	1.0 vvm by continuous injection 80% of initial saturation	12	5005 U/L 55.24 U/mg	Pinheiro et al. (2020)
*Marasmiellus palmivorus*	Stirred-tank (180 rpm)	Glucose and casein / peptone / glycerol/ mineral solution / potato broth	30% saturation	2.66	3420 U/mL	Schneider et al. (2018)
*Peniophora* sp.	Stirred-tank (150 rpm) Air-lift (without mechanical agitation)	Yeast extract/ peptone/ malt extract powder/ glucose/ artificial seawater/ CuSO_4_	vvm 2.0 vvm	5 7	2800 U/L 2300 U/L	Mainardi et al. (2018)

## Laccase immobilization: a sustainable strategy

Although laccases are numerous in origin, size and characteristics, the purpose for enzymatic immobilization is the same as for other enzymes. Immobilization encompasses the search for greater stability throughout storage and variations in operational parameters, as well as the possibility of recovery and reuse of these biocatalysts, which contributes to sustainable and cost-efficient processes.

Several experimental studies describe the immobilization of laccases with the subsequent application. However, few of the current review articles discuss immobilization methodologies used for the construction of these insoluble biocatalysts focusing on the advantages and disadvantages of these methodologies in different applications. Table 2 lists recent studies about laccases immobilization with an overview of the enzymes source, main characteristics, the immobilization methods, results obtained and the application of the immobilized biocatalyst.

**Table 2:** Current overview on laccases characteristics, immobilization techniques and related applications

Immobilization methods	Laccase source	Immobilization supports	Characteristics of the native enzyme			Results after immobilization	Results after application of the immobilized enzyme	Reference
			Optimum pH	Optimum temperature (°C)	Molecular weight (kDa)			
Entrapment- Physical retention of the enzyme in a network, usually an insoluble sol–gel porous matrix, followed by cross-linking	*Cyberlindnera fabianii*	Calcium (Ca-AIL) and copper alginate beads (Cu-AIL)	5.0	40	52	Increased storage stability after 21 days at 4 °C; Increased thermostability; After 8 reuses the residual activities were 36 and 40%, using ABTS as substrate	Degradation of 42.7% (Ca-AIL) and 39.1% (Cu-AIL) of bisphenol A (100 μM BPA) after 24 h	Olajuyigbe et al. (2019)
	*Trametes pubescens*	Calcium alginate beads and Crosslinking prior to entrapment in calcium alginate beads	3.0	40	–	Thermal and pH stability was improved; 40.3% of activity retention after 24 h at pH 9,0; Increased storage stability after 35 days at 4 °C; 70% of activity retention after 10 successive cycles of reuse	Degradation was greater than 99% of bisphenol A (20 mg/L) after 2 h	Lassouane et al. (2019)
	*Bacillus subtilis* MTCC 2414	Copper alginate beads	9.0	35	37	Thermal stability increase	81.72% degradation of Yellow GR dye (0.1% w/v) after 120 h	Narayanan et al. (2015)
	*Brevibacterium halotolerans* N11 (KY883983)	Alginate-gelatin	5.0	35	55	86.7% recovered activity; Increased pH and temperature stability; 65% of retention activity after 7 reuses using guaiacol as a substrate	Efficiency in decolorization of different classes of synthetic dyes	Reda et al. (2018)
	*Trichoderma harzianum* (HZN10)	Sol–gel matrix	6.0	50	56	93% immobilization efficiency; Increase in thermal, pH, and operational stability 82% of activity retention after 6 cycles of reuse using ABTS as a substrate	Dye decolorization: 100% of malachite green, 90% of methylene blue and 60% degradation of congo red (200 mg/L each) in the presence of 1-hydroxybenzotriazole (HBT) mediator	Bagewadi et al. (2017)
	*Cyathus bulleri*	Poly Vinyl Alcohol-based polymers crosslinked either by nitrate (PVA-nitrate) or boric acid (PVA-boric)	–	–	–	High immobilization yield, with 65 and 90% for PVA-boric and PVA-nitrate, respectively; - High resistance when exposed to high temperatures; 80% of activity retention after four months of storage at 4 °C	Batch decolorization of 95% of Basic Green 4 dye up to 20 cycles and 90% of Acid Red 27 up to 10 cycles (100 µM each) Continuous decolorization of 90% Acid Red 27 with a mediator (ABTS)	Chhabra et al. (2015)
	*Pseudomonas putida*	Nanofibers and carbon nanotubes (SWNT)	8.5	10	45	Increase in thermal, stability; After 5 cycles of reuse, 75% and 95% of the initial activity were maintained at 80 °C and 4 °C, respectively; Laccase activity was retained over 10 cycles of random freeze–thaw treatment	-	Mukhopadhyay et al. (2015)
Encapsulation- The enzyme is retained in spheres, such as semipermeable membranes thus preventing direct contact with the external environment	*Bacillus safensis* sp. strain S31	Alginate beads	5.0	30	–	–	Removed 95% of reactive black (10 mg/L) after 1 h	Siroosi et al. (2018)
Adsorption- Simple adsorption of the enzyme on support by bonds such as hydrophobic interactions, Van der Waals forces, hydrogen bonds, and ionic bonds	*Aspergillus oryzae*	Granular activated carbon	7.0	30	56	Thermal and pH stability was improved; 55% of activity retention after 20 cycles of reuse	Removal of more than 80% of sulfamethoxazole, carbamazepine, diclofenac and bisphenol A (each at 2.5 mg/L)	Nguyen et al. (2016)
	*Coprinus comatus*	Biochar	3.0	–	–	Immobilization yield of 64.2%; Thermal stability increase; 34% of activity retention after 7 cycles of reuses	71.4% removal of chlorinated biphenyl (0.04 g/L)	Li et al. (2018b)
	*Pleurotus ostreatus*	MANAE-agarose	5.0	50	60	Thermal stability increase; 70% of activity retention after 170 days of storage at 4 °C; In reuse, more than 90% of Bisphenol-A was degraded in the 15th consecutive cycle	Degradation of 90%Bisphenol-A (100 mg/L)	Brugnari et al. (2018)
	*Pleurotus ostreatus*	Porous acrylic carrier with octadecyl groups (C_18_)	–	–	–	94% of activity retention after 21 days (4 °C); Increase of thermal stability at 40 °C and 60 °C; 63% of activity retention, after four transformations for the synthesis of the dye in a continuous system	Production of orange dye (N15) through the transformation of 2-amino-3-methoxybenzoic acid	Wlizło et al. (2020)
	*Polyporus durus*	Nanoporous Zeolite-X	4.0	70	–	Immobilization yield of 83%; Thermal and pH stability was improved; After 7 cycles of decolorization, 100% of laccase activity was maintained	Decolorization of 100% of dyes Acid Blue 225 e Reactive Blue 19 (100 mg/L each) after 15 and 45 min, respectively	Wehaidy et al. (2019)
	*Rhus vernicifera*	Sepiolite (A); Sepiolite modified with chitosan (B); Sepiolite plus Cu(II) (C); Sepiolite modified with both chitosan and Cu(II) (D)	–	–	–	Improvement in enzymatic activity; Low desorption (< 10%) in all samples	–	Olshansky et al. (2018)
	*Trametes versicolor*	Metal-chelated chitosan-based copolymer nanoparticles	5.5	30	–	Thermal and pH stability was improved; 50% of activity retention after 8 cycles of reuse	Phenol degradation (20 mg/L) about 82% after 4 h, without a mediator; The addition of mediator ABTS improved phenol degradation (100% with 1 mM ABTS)	Alver and Metin (2017)
Chelation—The enzyme binds to the support by coordinated bonds, where the charged and polar amino acids such as histidine residues bind to metal ions	*Escherichia coli* (recombinant laccase)	Magnetic zeolitic imidazolate nanoparticles	–	70	65	75.7% recovered activity; Increased thermal and storage stability; The immobilized laccase retained 46.0% of the initial activity after 6 h at 80 °C; 87.1% of activity retention after 10 days of storage at 30 °C; Greater affinity (Km) to ABTS than the free enzyme	Complete decolorization of indigo carmine (25 mg/L) after five consecutive cycles	
Covalent bonds- Covalent binding of the enzyme to the support	*Aspergillus* sp.	Graphene oxide nanosheets	5.0	–	–	Immobilization yield of 64.6%; Decolorization of more than 75% of the dyes evaluated after 6 consecutive cycles	High efficiency of biodegradation of azo dyes in different concentrations	Kashefi et al. (2019)
	*Trametes versicolor*	Polyacrylonitrile-biochar composite nanofibrous membrane	4.5	30–40	–	Thermal, pH, and storage stability were improved; 71% of activity retention after one month of storage at 4 °C; 50% of activity retention after seven cycles of oxidation with ABTS	The biodegradation in the continuous mode of Chlortetracycline (200 ppb) exhibited 58.3% removal efficiency at the flux rate of 1 mL/h·cm^2^	Taheran et al. (2017)
	*Trametes versicolor*	Polyaniline electrodeposited onto a glassy carbon electrode/	3.0	–	–	Laccase biosensor has the highest current, demonstrating the highest catalytic ability for catechol oxidation than those without the enzyme	Biosensor for the detection of phenolic compounds (catechol)	Nazari et al. (2015)
	*Trametes versicolor*	Graphene oxide/CuFe_2_O_4_ nanocomposite	6.0	35	–	88% of activity recovery; Increased thermal and pH stability; 83% of activity retention after 30 days of storage at 4 °C	High efficiency in the synthesis of arylsulfonyl benzenediols (up to 91%); Conversion capacity of about 80% after 10 cycles of reuse	Rouhani et al. (2018)
	*Trametes versicolor*	Copper ferrite magnetic nanoparticles (CuMNPs) and ferrite magnetic nanoparticles (MNPs)	5.0	40	–	Activity recovery of 94.68 ± 0.92% and 89.78 ± 1.24% for CuMNPs and MNPs, respectively; Increased thermal and pH stability; At 70 °C, CuMNPs and MNPs showed relative activity of more than 70 and 60%, respectively; MNPs and CuMNPs retained more than 70% of its initial residual activity after 20 days (4 °C); After 6 cycles, the immobilized exhibited more than 70% of the initial activity	Delignification of plant biomass: CuMNPs: 43.28 ± 1.46% of lignin removal (160.6 mg lignin/g biomass); MNPs: 40.10 ± 1.35% (169.5 mg lignin/g biomass)	Muthuvelu et al. (2020)
	*Trametes versicolor*	Immobilized on the electrospun zein fiber (ceZL)				Relative activity of 92.76 ± 3.65% after immobilization; After 10 days of storage (4 °C), the ceZL remained higher than 81% of residual activity; The ceZL exhibited high relative activity between 4 and 40 °C	Optical biosensor to indicate the shelf life of food based on temperature (time–temperature indicator, TTI)	Jhuang et al. (2020)
	*Bacillus atrophaeus*	Magnetic-nanoparticles	5.5	35	31	Immobilization yield of 50%; The immobilized exhibited 60% of residual activity after 10 consecutive cycles with ABTS as a substrate; Greater stability at temperatures above 40 °C	Juice clarification: Reduction of total phenolic compounds (41–58%); Reduction of color and turbidity of 49–59% and 50–59%, respectively	Narnoliya et al. (2019)
Protein-inorganic hybrid nanoflower—This immobilization consists of a complex of enzymes cross-linked with metal ions with nanoflower (NF) morphology	*Trametes versicolor*	Cross-linked of laccase-Cu3(PO4)2∙3H2O hybrid NF	3.0	40	–	Yield and activity recovery of 78.1 and 204%, respectively; Improved catalytic efficiency, storage stability and greater solvent tolerance; CL-NF maintained 91.5% of the initial activity after 60 days of incubation at 4 °C; Residual activity of 92.3% after 10 reuse cycles	Decolorization of synthetic dyes (120 µg/mL each): bromophenol blue (41.2%), CBBR-250 (73.2%) and xylene cyanol (73.0%) after 48 h without mediators; the mediator (ABTS) increased the efficiency of decolorization	Patel et al. (2018)
CLEAs- In this method, there is no need for support. The immobilization occurs through cross-links between aggregated enzymes using bifunctional or multifunctional reagents	*Fomes fomentarius*	–	2.6	30	–	At pH 4.6 the immobilized enzyme retained twice the value of relative activity (60%) than the free one; Increased thermal and storage stability; 74% of activity retention after 70 days at 4 °C; After six reuse cycles, residual activity was about 50%	Decolorization around 90% of dyes malachite green (7 mg/L), bromothymol blue (50 mg/L) and methyl red (100 mg/L) after 10 h	Vršanská et al. (2018)
E-CLEAs- The soluble enzyme is aggregated by cross-linking and then entrapped within supports for the obtention of a better operational stability and reusability	*Trametes versicolor*	Entrapped cross-linked enzyme aggregate in mesoporous silica	4.5	–	–	Increased thermal, pH and storage stability; Retention of activity in solvents; 79% of activity retention after 20 cycles of reuse	Removal total of phenol (0.4 mM) in 40 min	Fathali et al. (2019)
M-CLEAs—Magnetic particles are bonded with CLEAs to provide enhanced mechanical stability. These biocatalysts are the easy separation from the reaction mixture and recycled by using a simple magnetic field	*Trametes versicolor*	Magnetically activated chitosan CLEAs (MAC-CLEAs)	4.0	40	–	The activity recovery of MAC-CLEAs reached 62.2%; –54% activity retention at pH 7.0; 67% of the activity at 60 °C; 32% of activity retention after 35 cycles of reuse	–Elimination of 13 pharmaceuticals (100 µg/L each): –After 6 h MAC-CLEAs effectively removed mefenamic acid (99%), acetaminophen (85%) and diclofenac (85%) and other compounds were partially removed, in some cases, ABTS was used as a mediator	Kumar and Cabana (2016)

Most studies in the literature cover immobilization and application of fungal laccases rather than bacterial and plant laccases. This fact may be related to the higher potential for oxireduction of fungal laccases in comparison to laccases from other sources, which allows these enzymes to be successfully applied in reactions with a variety of substrates. Besides, laccases expression is easily induced in fungi and the majority of fungal laccases are secreted to the extracellular space, which facilitates the steps of purification and subsequent immobilization (Baldrian 2006).

The choice of a particular immobilization method depends on the intrinsic properties of the enzyme. Different species can produce laccases with distinct biochemical properties (i.e., substrate affinity, molecular weight, redox potential, optimum temperature, and so on; see Sect. “Sources, production and directed evolution of laccases”), and intraspecies variation also occur, as laccase isoenzymes may present specific characteristics (Rivera-Hoyos et al. 2013). Though analogous protocols are used for laccases immobilization the results may differ from one enzyme to another. Therefore, each study reports very particular results of immobilization efficiency, which may be difficult to reproduce unless with the same enzyme (i.e., commercial enzyme) or similar enzymes (produced on a laboratory scale from an identified microorganism deposited in a culture collection and with comparable biochemical characteristics). For this reason, immobilization methods should be adapted or optimized (for evaluating parameters such as initial enzyme activity, pH, temperature and reaction time) respecting the properties of the native enzyme to achieve immobilization conditions with satisfactory results.

A wide variety of supports can be used in immobilization methods. Recently, Zdarta et al. (2018) and Daronch et al. (2020) addressed the most common options and also new materials with attractive characteristics to be explored in the processes. Alvarado-Ramírez et al. (2021), described in their review paper biologically-based materials and various agro-industrial waste supports as a potential alternative for laccase immobilization. The authors emphasize the need for the exploitation of agro-industrial wastes as enzymes supports could be useful in maintaining the overall cost-effective ratio of the entire process. And that the deployment of nanotechnological matrices for enzyme immobilization can improve the possibility of using these systems at an industrial scale.

Some materials used as enzymatic supports act as adsorbents and retain the reaction products through weak bonds (Wu et al. 2019). The adsorption in reactions using immobilized laccases has been previously reported (Kumar et al. 2014; Li et al. 2018a). As a result, accumulation of reaction product may occur near the active site, affecting the reaction kinetics or even changing the pH in the enzyme microenvironment. The retention of reaction products in the immobilization support can also make it difficult to quantify total protein or enzymatic activity by colorimetric methods.

During the immobilization process, the conditions of pH, temperature, agitation and chemical reactions cause substantial changes in the enzyme superficial microenvironment, in protein conformation and refolding. An inadequate choice of these conditions contributes to the lower enzyme activity and stability which is often observed after immobilization (Mohamad et al. 2015).

Alginate is a polymer commonly used in enzyme immobilization due to its biodegradability and cost-effectiveness. Laccases have been encapsulated in an alginate matrix combined with divalent ions such as barium, calcium, copper acting as crosslinking agents (Olajuyigbe et al. 2019). Bagewadi et al. (2017) immobilized the *Trichoderma harzianum* laccases in calcium alginate beads coated with chitosan and obtained an immobilization efficiency of 86%. The authors concluded that chitosan forms a polyelectrolyte complex with alginate thus increasing its mechanical properties. The stability of immobilized laccase in storage at 4 °C was enhanced in comparison to free enzyme. However, when analyzing the reuse of the immobilized laccase for 6 cycles, the catalytic efficiency was reduced to 66% at the end of the experiment, showing that continuous cycles negatively affected the stability of the immobilized enzyme.

A laccase from *T. versicolor* was immobilized by encapsulation in electrospun materials synthesized from poly (methyl methacrylate) and magnetite nanoparticles (Zdarta et al. 2020b). The immobilization yield was 100% for the encapsulated laccase and the kinetic parameter Vmax was improved after immobilization. Although the affinity (Km) of the immobilized enzyme for the substrate was lower than free form, the immobilization was considered effective due to Vmax. The lower affinity of the immobilized was related to the high porosity of the electrospun materials, which may have partially influenced diffusion limitations.

Adsorption on solid supports is the most common method among others proposed for enzymatic immobilization (Jesionowski et al. 2014). Different supports can be used for immobilization of laccases by adsorption (Table 2). Wen et al. (2019) immobilized laccase in bentonite-derived mesoporous materials and observed a 64% loss of activity after the 5th cycle of use, indicating leaching of the enzyme in the washing steps.

Recently, laccases have been successfully immobilized by adsorption on sepiolite modified with chitosan (Olshansky et al. 2018), on magnetic iron powder coated with chitosan (Patel et al. 2016), on cellulose modified with calcium nano carbonate (Li et al. 2018a), on magnetically modified bacterial cellulose (Drozd et al. 2018) and other supports. Among inorganic supports, laccases have been immobilized on hybrids of Zirconia-Silica and Zirconia-Silica with copper addition (Jankowska et al. 2019), on mesostructured silica (Zdarta et al. 2020a), on aluminum oxide (Kołodziejczak-Radzimska et al. 2020) and on mesoporous carbon nanospheres (Shao et al. 2019).

Laccases have been covalently immobilized on supports of different origins (Table 2), by different techniques. Yaohua et al. (2019) used as a strategy the prior encapsulation of ABTS mediator in cellulose spheres, which served as support for laccase covalent immobilization. This strategy, denominated as co-immobilization, provided a high catalytic activity for the immobilized enzyme, capable of degrading 99.7% of indole, a carcinogenic substance which was difficult to be degraded by the free enzyme in the absence of the mediator. In addition to catalytic efficiency, the derivative obtained good stability after 100 days of storage, degrading 96% of indole (15 mg/L) and with more than 60% of enzymatic activity after 10 cycles of reuse.

Bezerra et al. (2015) obtained promising results by covalently immobilizing *T.versicolor* laccases aminated in green coconut fibers pre-treated and activated by different protocols. Chemical amination of the enzyme was carried out by activating carboxylic groups (terminal carboxylic, Asp and Glu) with 1-ethyl-3- (dimethylamino-propyl) carbodiimide (EDAC) of its surface in the presence of ethylenediamine (EDA). The best results of immobilization were achieved with coconut fiber activated by glutaraldehyde (97–98%); however, the activation of the support with glyoxil groups resulted in a better thermal stability and recovered catalytic activity (59%).

Particularly for laccases, many efforts have been made to immobilize them by CLEAs (CLEAs-lac). *Trametes versicolor* CLEAs-lac were formed with (NH_4_)_2_SO_4_ (500 g/L) as a precipitant agent and chitosan (1 g/L), EDAC (50 mM) as crosslinker (Ba et al. 2014). CLEAs from *Fomes fomentarius* and *T. versicolor* laccases were produced by adding 75% (NH_4_)_2_SO_4_ and 50 mM glutaraldehyde (Vršanská et al. 2018) and from *Cerrena* spp. laccases with 30 mM glutaraldehyde and 80% (NH_4_)_2_SO_4_ (Yang et al. 2017c).

Immobilization by CLEAs eliminates the need for a previous purification step; the aggregates are easily formed by precipitation, followed by crosslinking of the enzyme (Cacicedo et al. 2019; Sheldon 2019). Cross-linking is advantageous because it is easily performed, generates aggregates with a high enzymatic load, stability, reusability and do not need a support, becoming inexpensive in comparison to other immobilization methods. The disadvantages are found in the conformational states of the enzyme during the catalytic process and the diffusional limitation of substrates and products due to aggregates morphology.

The authors Zerva et al. (2020) immobilized *Pleurotus citrinopileatus laccases* by CLEAs. The conditions were optimized resulting in a maximum of 72% residual activity. The most favorable parameters were 16 mg/mL of protein load, ammonium sulfate as precipitant and glutaraldehyde (100 mM) as crosslinker. The resulting CLEAs showed greater stability in the presence of solvents and at elevated temperatures compared to the free laccase preparation.

Immobilization by CLEAs can also occur by combining multiple enzymes, a process called combi-CLEAs (Sheldon 2019). Ba et al. (2012) simultaneously immobilized fungal laccases from *T. versicolor* and bacterial laccases from MetZyme in combi-CLEAs. In the free form, the *T. versicolor* laccase showed an optimal pH at 4.0, whereas the bacterial laccase showed maximum catalytic activity at alkaline pH of 8.0. The combination of active laccases in different pH levels presented greater versatility for the application of the immobilized biocatalyst in the treatment of industrial effluents. The ability of immobilized enzymes to withstand drying and storage effects has also been reported (Table 2), and these properties are promising for commercial use.

Currently, nanomaterials are highlighted as prominent supports for enzyme immobilization and constitute a group of new and viable matrices. Different nano-supports (organic and inorganic), as nanofibers, nanosheets, nanogels, nanotubes, etc. have been used for Lac-immobilization (Table 2), are applied to enhance stability and reusability of laccase, and posteriorly tested for diverse applications. Nanomaterials are highlighted as prominent supports for enzyme immobilization and constitute a group of new and viable matrices.

Lopez-Barbosa et al. (2020) reported on the use of the fungal laccase from *Pycnoporus sanguineus* CS43 immobilized on silica nanoparticles and entrapped in textile-based filters for the degradation of Congo Red dye. Six different types of filters were fabricated and tested in a continuous flow bioreactor. The results indicate removal efficiencies that approached 40% for enzymes immobilized on the more hydrophobic supports. Four-time more than obtained with similar filters treated only with free laccase (8%).

Nanoparticles with superparamagnetic properties and a high surface area also are promising nanomaterials for enzyme immobilization, as they can be modified to include reactive groups on their surface to bind enzymes such as laccases (Ba and Vinoth Kumar 2017). Besides, they are used to magnetize other supports with an enzyme previously bound and also form magnetized aggregates (magnetized CLEAs) (Yang et al. 2017b). The easy separation of the enzyme from the reaction mixture using a simple magnetic field is an additional and attractive feature, avoiding the use of ultracentrifugation and/or nanofiltration with special membranes and therefore reducing application costs (Gupta et al. 2011).

## Scientific production, current applications and patents

### Scientific production and current applications

The application of laccases for biotechnological purposes is undoubtedly a promising area of research. The number of scientific publications recovered from “Web of Science” platform using the terms “laccase”, “laccase immobilization”, “laccase immobilized”, “laccase production” and “laccase bioreactor” in the last five years were 3,916, 830, 787, 1008 and 92 respectively.

The number of publications demonstrates the interest not only in laccases production but also in the development of immobilization methods for the application of these biocatalysts. Most cited applications of laccases in biotechnological processes include their use in textile industries and dye degradation, degradation of environmental pollutants, biosensors, delignification, food processing, paper and pulp industry and polymer synthesis.

In textile industries, laccase enzymes can be applied not only in the treatment of effluents with dyes but also in textile bleaching. Laccases may also be used in combined processes of dye discoloration and textile bleaching (Iracheta-Cárdenas et al. 2016; Zhang et al. 2018, 2020a, b; Unuofin 2020). Most studies to date have reported the use of laccases (free or immobilized) for biodegradation of synthetic dyes in aqueous solutions (using mainly water as solvent) or in simulated effluents, but few authors have tested laccases for treatment of real textile effluents (Sondhi et al. 2018; Navada and Kulal 2020). This is a necessary gap to be filled for improved application of laccases in textile industries.

In pulp and paper industries, an alternative to the chemical bleaching process is bio-bleaching with enzymes (Aslam et al. 2016; Nathan et al. 2018). Laccases produced from white-rot fungi can degrade lignin and increase paper brightness. Bio-bleaching efficacy with laccases can also be improved with the use of synthetic mediators, which may be replaced by natural ones to reduce the toxicity of the process (Ozer et al. 2020). The use of a mixed cocktail of ligninolytic and hemicellulolytic enzymes may result in a significant improvement of pulp properties and reduction of chlorine (Angural et al. 2020a, b).

In the food industry, laccases can be applied as biomarkers/biosensors and enzymatic modifiers of dough. The wine industry has used laccases as biomarkers to detect contamination by *Botrytis cinerea* in grape must (Zimdars et al. 2017). Electrochemical laccase biosensors have been used as efficient alternatives for the detection of phenolic compounds because of their sensitivity and selectivity (Bilir et al. 2016; Medina-Plaza et al. 2016; Gonzalez-Anton et al. 2017). In baking, laccases can be used to improve the resistance of gluten structures in doughs and also to produce gluten-free flours (Serventi et al. 2016; Manhivi et al. 2018). Laccases can also be used for manufacturing an enzymatic time–temperature indicator (TTI) of food products (Jhuang et al. 2020).

In the environmental field, studies describing the use of laccases for enzymatic treatment/biodegradation of many different organic pollutants in water and soil have increased in number. Laccases act on the biodegradation of contaminants such as phenolic compounds, synthetic dyes, pesticides, drugs, personal care products, hormones, and others (Bilal et al. 2019). A recent trend in scientific literature is the evaluation of immobilized laccases in bioremediation/biodegradation studies (Brugnari et al. 2020). Biocatalytic properties of laccases immobilized in nanomaterials are described in biodegradation of chlorophenol (Zhang et al. 2020b), diclofenac and naproxen (Zdarta et al. 2019), tetracycline (Zdarta et al. 2020b) and others. Besides, decontamination of soil containing 2,4-dinitrophenol, using free and immobilized laccases on glutaraldehyde-coated montmorillonite and zeolite minerals, demonstrated high efficiency, 98.6% after 16 h incubation (Rahmani et al. 2020).

Other important and current applications of laccases include: pretreatment of lignocelluloses (mainly agroindustrial wastes or byproducts) for production of biofuels (Deng et al. 2019; Matei et al. 2020), biocatalysts in organic synthesis of bioactive compounds (Kudanga et al. 2017; Su et al. 2018) and laccase mediated grafting of lignocelluloses and synthetic polymers (Slagman et al. 2018).

Publications about laccase production increased over the years, with 201 publications in 2017, 236 in 2020 and 121 until July 2021. The rising number of these publications demonstrates interest in these enzymes for different processes. The results obtained for the keywords “laccase bioreactor” also encompass bioreactors using laccases for the treatment of wastewater. Using the refine results, with the word “scale”, the results were reduced to 28 publications, demonstrating that scaling up of laccase production is still a new field in research and is still on the rise.

As previously mentioned, bioreactors are the main unit operations used to produce laccase when the scale-up to industries is desired. For this, the authors aim to optimize the production process of laccases on a larger scale, varying process parameters (Pinheiro et al. 2020; Abdelgalil et al. 2021). The authors Nikolaivits et al. (2021) evaluated the production of laccase in the presence of copper ions in a 12 L bioreactor, looking for laccases with greater bioremediation capacity when produced in the presence of mediators aiming to study the laccase production on a larger scale. Ardila-Leal et al. (2019) also evaluated the composition of the medium in the scale-up of laccase enzyme production in 10 L bioreactors, increasing the scale and improving the enzyme production through the optimization of parameters.

### Commercial laccases and patents

The use of laccases in industrial processes is considered ecologically viable and fits the concept of circular economy, according to the slogan “reduce-reuse-recycle”. A report on the worldwide market for industrial applications of enzymes has estimated a billion-dollar growth in the area over the next five years, projecting a value of US$ 8.7 billion in 2026, at a compound annual growth rate (CAGR) of 6.3% for the period 2021–2026 (BBC Research 2021). Laccases, due to their versatility for use in different segments, represent a potential target for the global enzyme market. In 2020, the global laccase market size was of approximately US$ 3 million, and it is expected to reach US$ 4 million by the end of 2027, with a CAGR of 4.3% between 2021 and 2027 (Precision Reports 2021).

Promising results have been reported in the environmental field (Darvishi et al. 2018; Zhang et al. 2020a; Imam et al. 2021), food industry (Giacobbe et al. 2018; Mayolo-Deloisa et al. 2020) and cosmetics (Shin et al. 2019), pharmaceutical industry.

The circular economy, when compared to the linear economy, is considered more advantageous and within future expectations of the market (Mayolo-Deloisa et al. 2020). Laccases have been marketed for decades, and one of the oldest commercial products with the enzyme was launched in 1996 by Novozymes (Novo Nordisk, Denmark): DeniliteI™, the first industrial laccase, based on a thermostable recombinant *M. thermophila* laccase expressed in *A. oryzae*, for fabric bleaching (Rodriguez-Couto 2012). Since then, new formulations and applications have been investigated, patented and applied in the textile industry for denim finish (DeniLiteII™ and Zylite), as well as in other areas such as pulp bleaching (Lignozym^®^ Process), paper pulp delignification (Novozym^®^ 51,003), to prevent contamination in cork stoppers (Suberase^®^) (Rodríguez-Couto 2019) in the food industry, colour enhancement in tea, etc. LACCASE Y120 Amano Enzyme USA Co. Ltd, Brewing Flavourstar Advanced Enzyme Technologies Ltd. (India) (Osma et al. 2010; Mayolo-Deloisa et al. 2020).

Among the applications already consolidated industrially, the textile industry is the largest field. The benefits of the application are relevant and include fewer chemicals, energy and water-saving features, thus contributing to a more environmentally friendly process. Laccase is also used in situ to convert dye precursors for better, more efficient fabric dyeing (Rodríguez-Couto 2015). The treatment of wastewater polluted with indigo and other dyes has also been investigated using commercial laccases. However, the application of laccases in effluents in their free form is not usually carried out, as they present some inconveniences, such as their rapid inactivation in these waters caused by salts and solvents. Thus, enzymes in the free form are not recovered and consequently increase the process cost. For these reasons, the immobilization of laccases in various materials, which improve their stability, has gained great relevance in this area (Gasser et al. 2014). In the last decade, several patents have reported the treatment of wastewater with immobilized laccases, either for the removal of dyes or for other pollutants, such as those from the pharmaceutical and food industry, indicating a future perspective for the real application of these enzymes in this field as well.

So far, most laccases produced on a commercial scale are of fungal origin. As mentioned earlier in this paper, the cost-effectiveness of producing laccase of bacterial or plant origin is still unattractive from a commercial point of view. However, advances in this field have been observed as the Finnish company MetGen recently commercialized an industrial alkaliphilic bacterial laccase MetZyme^®^, which is an integral part of METNIN™ lignin fractionation technology to produce highly reactive depolymerized lignin in a very sustainable and efficient manner. Thus, studies into the use of bacterial laccases on an industrial scale have been performed in the last years.

Currently, there are six major suppliers of laccases all over the world, including Novozymes, DuPont, Amano Enzyme, Yiduoli, Sunson and Denykem. Novozymes is the world leader in this industry and DuPont is the second-largest producer. Market concentration in this industry is the high and total market share of those six suppliers are 98% (Precision Reports 2021).

**Fig. 3:**
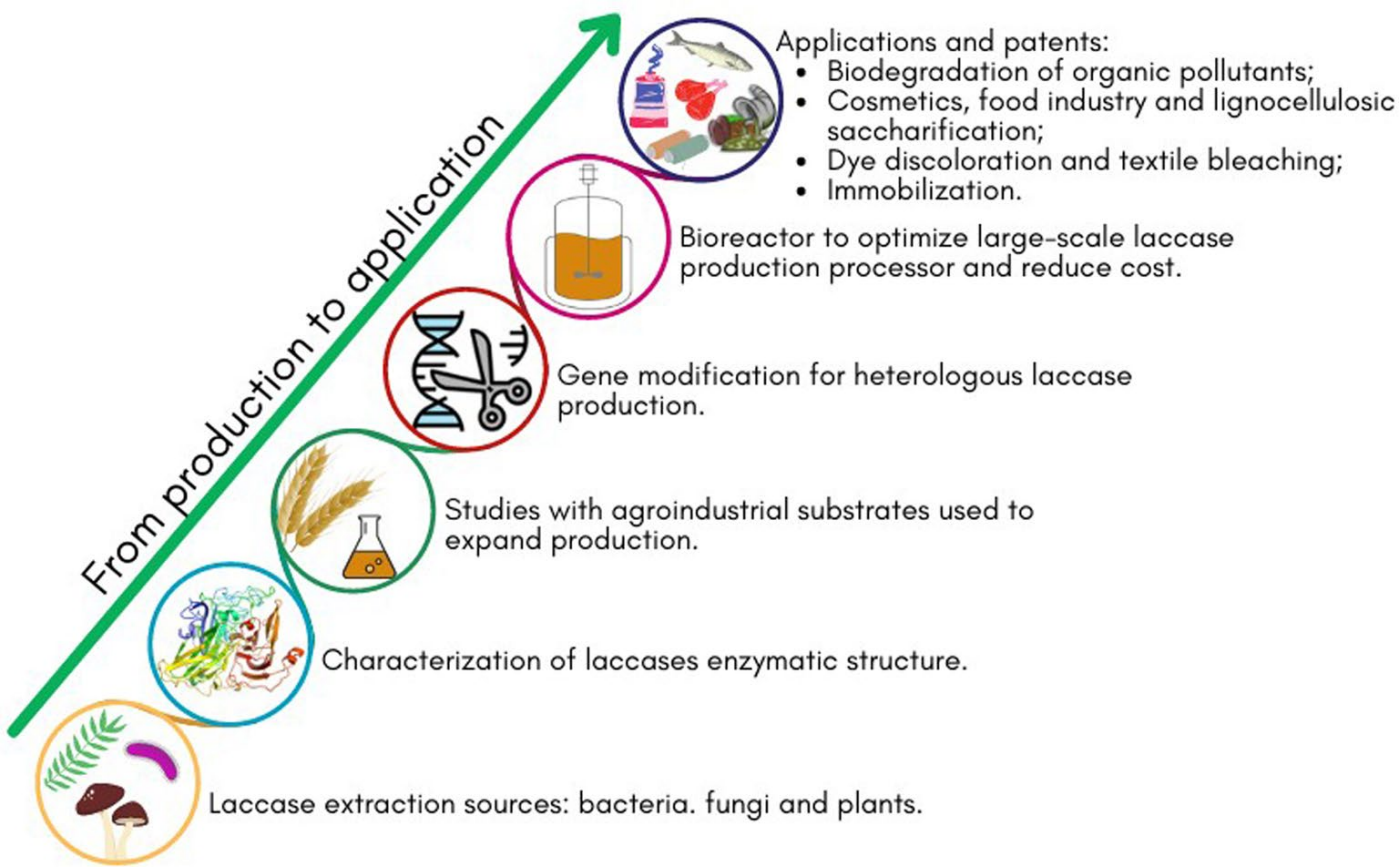
Main steps to achieve large-scale laccase production: laccase extraction sources, selection of suitable and low-cost substrates, enzyme characterization, genetic modifications for improved enzymatic production and bioreactor cultivation. These stages are fundamental for laccase commercialization and application. (Laccase structure available on Gecco Groningen Enzyme e Cofactor Collection, September 2021)

Despite all these findings, commercial production of laccases remains relatively static when compared to the production of several other enzymes, such as cellulases, amylases, and proteases. Amylase enzymes constitute the largest segment of the biofuel enzyme industry, with revenues of $226.6 million in 2011. Cellulases are the second leading category of enzymes for biofuels, with revenues of $155.8 million in 2011. Lipases had a revenue of $39.6 million in 2011(BBC Research 2013). As mentioned earlier, the price of laccase may vary according to different production conditions. Some researchers have been trying to find alternatives to make these enzymes more accessible on an industrial scale (Fig. 3). Table 3 shows the price of different commercial laccases and the price of laccases estimated by the researchers in their scale-up experiments. Cost savings can be expected with increased enzyme production.

**Table 3:** Cost of different commercial laccases and laccases produced by researchers in scale-up experiments

Product Brand/ Reference	Enzyme source	Amount	Price [€]	Activity (U) or specific activity (U/g)	Specific cost (€/kU)		^1^Final specific cost (€/kU)
Sigma Aldrich/ 38,429	Laccase from *Trametes versicolor*	10 g	€ 534,00	^a^500 U/g	106.8	83.30	
Sigma Aldrich/ 40,452	Laccase from *Agaricus bisporus*	1 g	€ 671,00	^a^4000 U/g	167.7	130.85	
Sigma Aldrich/ L2157	Laccase from *Rhus vernicifera*	10,000 UNITS	€ 340,00	^b^10000U	34.0	30.94	
Sigma Aldrich/ SAE0050 Synonym Novozym^®^ 51,003	Recombinant laccase from *M. thermophila* expressed in *Aspergillus oryzae*	250 mL	€255,00	^b^1000U/g	1.02	0.93	
Pezzella et al. (2017)	Laccase from *Pleurotus ostreatus* expressed in yeast *Pichia pastoris* rPOXA1b	–	–	–	0.34	0.34 (2017) ^2^0.56 (2021)	
Lú-Chao et al. (2018)	Laccase from *Ganoderma Lucidum*	–	–	–	1.08	1.08 (2018) ^2^1.52 (2021)	

The increased demand for specific and stable biocatalysts as alternatives to chemical catalysts and/or chemical compounds has encouraged the industrial sector and research centers to deposit many patents related to immobilized laccases and their applications (Table 4).

**Table 4:** Patents deposited by different inventors/institutions concerning different applications of laccases

Title of patent	Applications	Laccase form used	Immobilization method		Current Assignee	Inventors and Patent number
Co-immobilized enzyme and preparation method and application there of	Wastewater treatment	Immobilized	Immobilization is carried out by combining an embedding method and a physical adsorption method		Xiamen University of Technology, 2019	Haiyan F, Yuezheng J, Yicheng W, Hongda Z, Zheng L, Jianzhen L, Dongyang W (CN110628756)
Hollow mesoporous carbon immobilized laccase and preparation method	Removal ciprofloxacin dye wastewater	Immobilized	Physical adsorption method or a covalent binding method (or laccase carries out agglomeration in the inner cavity)		Hunan University, 2018	Zhifeng L, Binbin S, Guangming Z, Zhigang L, Yang L, Yujie J, Yilin T (CN107893066)
Immobilization of enzymes	Organic synthesis	Immobilized	Adsorption immobilization		Novozymes AS, 2016	Mazeaud I, Poulsen PBR, Christensen MW, Brask J (US 9303.256 B2)
Method for preparing MGO-laccase	Decolorization of a malachite green dye	Immobilized	Covalent linkage		Nanjing Ligong Shuifu Environmental Protection Technology; Nanjing Forestry University, 2019	Weichuan Q, Hai L, Bo F (CN110195053)
Method for utilizing laccase to dispose printing and dyeing wastewater	Disposal of printing and dyeing wastewater	Immobilized	Active carbon granule immobilization, the resin immobilization and the epoxidation PVA immobilization		Fuzhou University, 2019	Jie Y, Zhiwei L, Juan L, Xiuyun Y (CN109652389)
Method of degradation and inactivation of antibiotics in water by immobilized enzymes onto functionalized supports	Degradation of the cyclin family (tetracycline, oxytetracycline (OTC) and chlortetracycline) whose penicillin (amoxicillin), cephalosporin (cefdinir) and carbapenem (imipenem)	Immobilized	Covalent linkage or gel entrapment		Luxembourg Institute of Science and Technology (LIST), 2020	Bonot S, Cauchie HM (CN10538443)
Co-crosslinking immobilization method of laccase	Improve catalytic capacity	Immobilized	Co-crosslinking immobilization		University of Nottingham Ningbo China, 2020	Jiaqin W, Ruifeng Z, Yan L, Tonghu X, Nengbing L (CN110760502)
Preparation method of ultrafiltration membrane with *in-situ* immobilization of laccase and treatment method of phenolic wastewater	Wastewater treatment with phenolic compounds	Immobilized	In situ laccase immobilized ultrafiltration membrane		Anhui Polytechnic University, 2018	Hai T, Chen Z, Qiang L, Haonan Z, Baoyu Q, Jianping X (CN109012219)
Process for isolating ligninolytic-enzyme-producing microorganisms from agro-industrial waste, process for producing lignin derivatives, a cosmetic hair-dying agent and enzymes, a cosmetic hair-straightening agent and a cosmetic hair-dying composition	Cosmetic composition for hair dyeing	Free-form	–		Rosa Maria Teixeira Tage Biaggio, 2018	Rosa Maria Teixeira Tage Biaggio (WO2019227191A1)
A novel hair bleaching method	A hair bleaching method combining laccase pretreatment with oxygen bleaching	Free-form	–		Nantong University, 2018	Jia Weini, Mao Qinghui, Qu Jiangang, Wang Haifeng, Wang Wei; Zhang Chencheng, Zhang Xiaoli (CN108570849A)
Modified laccase soaping agent for cotton fabric soaping and preparation method thereof	A modified laccase soaping agent for cotton fabric soaping	Free-form	–		Suzhou Dabang Textile CO LTD, 2019	Zhang Zhanhui (CN111593588A)
Detergents with improved detergent power, containing at least one laccase	Use of specific laccases during the washing of textiles	Free-form	–		Henkel AG & CO KGAA, 2018	Mussmann Nina, O'connell Timothy, Herbst Daniela, Prüser Inken, Berger Ralf G, Linke Diana, Behrens Christoph (EP3303572)
Novel laccase from *Ganoderma lucidum* capable of enhancing enzymatic degradation of lignocellulolytic biomass	Degradation of lignocellulolytic biomass	Free-form	–		Danmarks Tekniske Universitet, 2018	Anna Sitarz, Jørn Dalgaard Mikkelsen, Anne Meyer, Mateusz Lezyk (US20180030420)
Laccases for bio-bleaching	bio-bleaching and decolorization of wood pulp	Free-form	–		Basf Enzymes LLC, San Diego, CA (US); BASF SE, Ludwigshafen am Rhein (DE), 2016	Janne Samuli KeroVuo, Sylke Haremza, Oliver Koch, Tilo Habicher, Dan E. Robertson, Grace Desantis, Ryan McCann, Peter Luginbuhl (US20160237411)

Data were obtained from WIPO and EPO databases

New industries and research centers related to enzymes production/application are located mainly in Asia, which has presented growth in these sectors even in the midst of global crises, especially because less bureaucracy, higher availability of supplies and fast industrial production with a large labor force and low cost (Piscitelli et al. 2016). In contrast, in Europe, small companies were founded in this field due to the high demand for alternative biocatalysts and clean technologies, which aim to offer new formulations for laccases and ideal conditions for their applications (Pezzella et al. 2015).

In a search for patents at WIPO (World Intellectual Property Organization, https://www.wipo.int) the number of documents found within terms “laccase” and “laccase immobilization or immobilized laccase” in the last 5 years were 6,344 and 711, respectively. The number of patents related to immobilized laccases between the years 2016–2020 presented an annual average of 142.2 deposits, with higher results in 2019. When assessing the number of patents in the last five years related to the term “laccase” in general, the United States is the country that has the largest number of deposits in this area in recent years. China is the country that most filed patents related specifically to the immobilization of these enzymes, followed by the USA. Moreover, according to the same databases, the companies NOVO NORDISK A/S, NOVOZYMES A/S, and L'OREAL were, in this order, those most related to patents of immobilized laccases, and the companies NOVOZYMES A/S, PROCTER & GAMBLE and L'OREAL were the ones that most deposited patents with laccase in general.

In EPO (European Patent Office, https://www.epo.org) the total number of patents found with the search terms “laccase”, “laccase immobilization or immobilized laccase” were 10,528 and 184, respectively. The number of patents related to immobilized laccase in the period between 2016 and 2020 presented an annual average of 36.8, with higher results in 2019. According to the data presented at the platform in the period, China remained as the country that most registers and leads the world patent indicators with laccase, followed by the United States. The companies NOVOZYMES AS, PROCTER & GAMBLE, DANISCO US INC are most applicants. China and USA are also the leading countries in patents applications about laccase immobilization, and the predominant companies are CHINA PETROLEUM & CHEM CORP and SINOPEC DALIAN RES INST PETROLEUM & PETROCHEMICALS.

These results indicate the magnitude of laccases applications and the increase in the patent growth curve aimed at this area for the following years.

## Conclusions

Laccases are remarkable biocatalytic tools with applications in numerous biotechnological processes. The production and commercialization of laccases is part of a million-dollar market that is still expanding. Although extensive, laccase research is still mostly performed on the laboratory scale or in controlled laboratory conditions. Therefore, emphasis should be placed on increasing studies of scaling up processes for production, economic analysis, and further application of laccases. In addition, testing laccases in real or simulated situations (e.g., real textile effluents) will effectively determine the possibility of using these enzymes on larger scales. These issues represent a challenge for innovative research on the use of laccases as sustainable biocatalysts.

Immobilization of laccases have increased the viability for industrial use of these enzymes which acquire characteristics such as improved stability and the possibility of easy recovery and reuse. Several efficient methods for laccase immobilization are available in scientific literature, however, it is not possible to define a universal protocol for all laccase isoforms produced from diverse sources under different conditions. The success of a particular method depends on the intrinsic properties of a particular laccase. Besides, some immobilization methods have limitations that restrict commercial applications. Enzymatic leaching processes, loss of catalytic activity, limitations of mass transfer between enzyme and substrate, difficulty in quantifying the immobilized enzyme or the use of expensive supports are examples of barriers that need to be overcome.

Free and immobilized laccases are attractive in different lines of research and the expressive number of scientific publications documented in the last 5 years reinforces the versatility of these biocatalysts. The growing demand for the use of these enzymes is also reflected in the number of patents deposited by industries and research centers. However, more work is necessary to demonstrate the economic and green feasibility for the real application of laccases in different fields.

## Data Availability

The datasets generated during and/or analysed during the current study are available from the corresponding author on reasonable request.
